# Clinical Significance of Tumor Markers for Advanced Thymic Carcinoma: A Retrospective Analysis from the NEJ023 Study

**DOI:** 10.3390/cancers14020331

**Published:** 2022-01-11

**Authors:** Tomoyasu Mimori, Takehito Shukuya, Ryo Ko, Yusuke Okuma, Tomonobu Koizumi, Hisao Imai, Yuichi Takiguchi, Eisaku Miyauchi, Hiroshi Kagamu, Tomohide Sugiyama, Keisuke Azuma, Yukiko Namba, Masahiro Yamasaki, Hisashi Tanaka, Yuta Takashima, Sayo Soda, Osamu Ishimoto, Nobuyuki Koyama, Kunihiko Kobayashi, Kazuhisa Takahashi

**Affiliations:** 1 Department of Respiratory Medicine, Juntendo University Graduate School of Medicine, Tokyo 113-8421, Japan; t-mimori@juntendo.ac.jp (T.M.); kztakaha@juntendo.ac.jp (K.T.); 2 Division of Thoracic Oncology, Shizuoka Cancer Center, Shizuoka 411-8777, Japan; 3 Department of Thoracic Oncology and Respiratory Medicine, Tokyo Metropolitan Cancer and Infectious Disease Center Komagome Hospital, Tokyo 113-8677, Japan; yokuma@ncc.go.jp; 4 Department of Thoracic Oncology, National Cancer Center Hospital, Tokyo 105-0045, Japan; 5 Department of Hematology and Medical Oncology, Shinshu University School of Medicine, Matsumoto 390-8621, Japan; tomonobu@shinshu-u.ac.jp; 6 Division of Respiratory Medicine, Gunma Prefectural Cancer Center, Ota 373-8550, Japan; m06701014@gunma-u.ac.jp; 7 Department of Respiratory Medicine, Saitama Medical University International Medical Center, Hidaka 350-1298, Japan; kagamu19@saitama-med.ac.jp (H.K.); kobakuni@saitama-med.ac.jp (K.K.); 8 Department of Medical Oncology, Chiba University Graduate School of Medicine, Chiba 260-8677, Japan; takiguchi@faculty.chiba-u.jp; 9 Department of Respiratory Medicine, Tohoku University Hospital, Sendai 980-8574, Japan; miyauchi@rm.med.tohoku.ac.jp; 10 Division of Thoracic Oncology, Tochigi Cancer Center, Utsunomiya 320-0834, Japan; tomsugiy@tochigi-cc.jp; 11 Department of Pulmonary Medicine, Fukushima Medical University, Fukushima 960-1295, Japan; k-kenya@fmu.ac.jp; 12 Department of Respiratory Medicine, Juntendo University Urayasu Hospital, Urayasu 279-0021, Japan; yknanba@juntendo.ac.jp; 13 Department of Respiratory Disease, Hiroshima Red Cross & Atomic-Bomb Survivors Hospital, Hiroshima 730-8619, Japan; myamasanjp@hiroshima-med.jrc.or.jp; 14 Department of Respiratory Medicine, Hirosaki University Graduate School of Medicine, Hirosaki 036-8563, Japan; h-tanaka@cc.hirosaki-u.ac.jp; 15 Department of Respiratory Medicine, Faculty of Medicine, Hokkaido University, Sapporo 060-8648, Japan; y.takashima@huhp.hokudai.ac.jp; 16 Department of Pulmonary Medicine and Clinical Immunology, Dokkyo Medical University, Tochigi 321-0293, Japan; s-s-sayo@dokkyomed.ac.jp; 17 Department of Pulmonary Medicine, Sendai Kousei Hospital, Sendai 980-0873, Japan; oishimoto@gmail.com; 18 Department of Pulmonary Medicine, Okino Medical Clinic, Miyagi 984-0831, Japan; 19 Division of Pulmonary Medicine, Clinical Department of Internal Medicine, Jichi Medical University Saitama Medical Center, Saitama 330-8503, Japan; nkoyama@saitama-med.ac.jp; 20 Department of Pulmonary Medicine, Saitama Medical Center, Saitama Medical University, Saitama 350-8550, Japan

**Keywords:** advanced thymic carcinoma, prognostic factor, tumor marker, neuron-specific enolase, squamous cell carcinoma antigen

## Abstract

**Simple Summary:**

Advanced thymic carcinoma (ATC) is rare. Owing to its rarity, there is limited information on the prognostic factors, and the optimal serum tumor markers are also unknown. We conducted a multi-institutional retrospective study of patients with ATC. In this study, we collected data on patient characteristics, progression-free survival (PFS), overall survival (OS), and tumor marker values, and investigated the relationship between tumor marker values and PFS/OS. We found that the neuron-specific enolase (NSE) level may be a useful prognostic tumor marker for ATC, regardless of histology. The findings of the analysis limited to squamous cell carcinoma suggested that the NSE and squamous cell carcinoma antigen levels may be useful prognostic factors.

**Abstract:**

The optimal tumor marker for predicting the prognosis of advanced thymic carcinoma (ATC) remains unclear. We conducted a multi-institutional retrospective study of patients with ATC. A total of 286 patients were treated with chemotherapy. Clinicopathological information, including serum tumor markers, was evaluated to determine the overall survival (OS) and progression-free survival (PFS). The carcinoembryonic antigen, cytokeratin-19 fragment, squamous cell carcinoma (SCC) antigen, progastrin-releasing peptide, neuron-specific enolase (NSE), and alpha-fetoprotein levels were evaluated. In the Kaplan–Meier analysis, the OS was significantly shorter in the patients with elevated NSE levels than in those with normal NSE levels (median, 20.3 vs. 36.8 months; log-rank test *p* = 0.029; hazard ratio (HR), 1.55; 95% confidence interval (CI), 1.05–2.31 (Cox proportional hazard model)); a similar tendency regarding the PFS was observed (median, 6.4 vs. 11.0 months; log-rank test *p* = 0.001; HR, 2.04; 95% CI, 1.31–3.18). No significant differences in the OS and PFS were observed among the other tumor markers. In both univariate and multivariate analyses of the patients with SCC only, the NSE level was associated with the OS and PFS. Thus, the NSE level may be a prognostic tumor marker for thymic carcinoma, regardless of histology.

## 1. Introduction

Thymic epithelial tumors, including thymoma and thymic carcinoma (TC), are rare intrathoracic malignancies that occur in the prevascular mediastinum [1]. TC is an even rarer malignancy, with an annual incidence of 0.13 cases/100,000 population, and accounting for ~15% of all thymic epithelial tumors [2,3]. It has a propensity to invade the surrounding tissues and metastasize, and one-half to two-third of all patients with TC are diagnosed with locally advanced or metastatic disease [4,5,6]. In the stage classification for TC, both the Masaoka staging system [7] and World Health Organization (WHO) TNM classification [8] are used, although the Masaoka staging system is used more frequently. The 5-year survival rate of patients with TC is 90% for Masaoka stage 1–2 and 30% for stage 3–4. This indicates that the prognosis of advanced thymic carcinoma (ATC) is poor as it progresses [9].

In patients with ATC, systemic chemotherapy is the standard of care, but the disease is so rare that an optimal regimen has not yet been established. Based on the results of small phase II trials and retrospective studies, combination chemotherapy, platinum-based doublets (e.g., carboplatin and paclitaxel [6,10], cisplatin or carboplatin and etoposide for thymic neuroendocrine carcinoma [11,12,13]), and other multidrug regimens (e.g., doxorubicin, cisplatin, vincristine, and cyclophosphamide [ADOC] [14,15] and cisplatin, doxorubicin, and vincristine [CAP] [16]) are selected for patients with ATC in clinical practice. The efficacy of these regimens are modest; however, the response to chemotherapy and outcomes vary considerably among patients. Therefore, predictive and prognostic biomarkers are required for ATC patients receiving chemotherapy.

Although the prognostic factors in patients with ATC treated with chemotherapy remain unclear, there have been a few reports on the prognostic factors in patients with TC. In these limited reports, the Masaoka stage [5,17,18], age [19], sex [5,20], race [5], Karnofsky performance status (PS) [18], histology [18], degree of resection [18,21], presence of radiotherapy [18,22], white blood cell count [23], and lactate dehydrogenase level [23] were reported as prognostic factors. However, most patients included in these studies had early stage diseases that can be treated locally, and data with advanced stages are limited.

For tumor markers, there are no relevant reports in ATC, although tumor markers are used clinically in many other carcinomas. For example, in lung cancer, serum tumor markers, including the carcinoembryonic antigen (CEA) [24,25,26,27], cytokeratin-19 fragment (CYFRA) [25,26,28], squamous cell carcinoma (SCC) antigen [27], progastrin-releasing peptide (ProGRP) [29], and neuron-specific enolase (NSE) [26,27,28,30] levels, have been considered to be predictive or prognostic, although no consensus has been reached on how to use them in daily clinical practice. It remains unclear whether there are optimal tumor markers for ATC.

We previously reported the results of a multi-institutional retrospective study (NEJ023 study) of patients with ATC, and found that using the Masaoka staging system and a history of volume reduction surgery may be a prognostic factor [31].

In this study, we collected clinicopathological information, including tumor markers, from the NEJ023 study, and analyzed the overall survival (OS) and progression-free survival (PFS). The study aimed to determine whether tumor markers can be prognostic factors and which of them is correlated with prognosis the most. This study was registered with the University Hospital Medical Information Network Clinical Trials Registry (identifier: UMIN000015649).

## 2. Materials and Methods

### 2.1. Patient Cohort

The details of the study design and results of first-line chemotherapy in ATC patients have been previously published [31]. In this observational multicenter study, we retrospectively reviewed the medical records of patients who received chemotherapy between 1995 and 2014. All institutions belonging to the North East Japan Study Group were invited to participate. The inclusion criteria for this study were (a) a histologic diagnosis of TC at local institution; (b) advanced-stage or recurrent disease treated with palliative-intent pharmacotherapy.

### 2.2. Data Analysis

Data were obtained from 324 consecutive patients from 40 institutions. Thirty-seven patients who did not meet the eligibility criteria and one patient with missing data were excluded. Finally, 286 patients who received first-line chemotherapy were included in the analysis. The following details were extracted from the medical records of the patients: age, sex, Eastern Cooperative Oncology Group PS, Masaoka–Koga stage [32], WHO TNM stage, histology [8], date of death or last follow-up, regimens of first-line chemotherapy (platinum-based doublet, other multidrug regimens or single agents), duration of chemotherapy, and efficacy of chemotherapy. Histologic subtypes were determined according to the 2004 WHO classification [8] in each institution. PFS data collection was not mandatory in this study; therefore, only cases for which data could be collected were analyzed.

Furthermore, we collected data on tumor markers before the start of first-line chemotherapy. In the recurrence cases, tumor markers taken at the time of recurrence were collected. In the setting of the study, there was no provision for mandatory measurement of tumor markers, and data on cases that were measured voluntarily were collected. The CEA, CYFRA, SCC antigen, NSE, ProGRP, and alpha-fetoprotein (AFP) levels were evaluated as the tumor markers. For each tumor marker, the normal levels were defined as follows, with reference [26,33,34,35,36,37] to the values commonly used in other carcinomas: CEA level, ≤5.0 ng/mL; SCC antigen level, ≤1.5 ng/mL; CYFRA level, ≤3.5 ng/mL; ProGRP level, ≤80 pg/mL; NSE level, ≤10 ng/mL; and AFP level, ≤10 ng/mL; values above these limits were defined as elevated.

The PFS was evaluated by investigators based on the Response Evaluation Criteria in Solid Tumors version 1.1 [38].

The primary purpose of this study was to investigate tumor markers that correlate with OS, and for other purposes, the relationships between tumor markers and PFS were investigated. The effect of each tumor marker on the OS and PFS was examined using a univariate analysis, dividing patients according to normal and elevated values. Thereafter, when a significant result was obtained in the univariate analysis, we performed a multivariate analysis including the prognostic factors examined in the previous report [31].

Generally, the most common histological type is SCC, which accounts for 70–80% of the total cases [8,19]. An analysis limited to the patients with SCC only was performed in addition to the entire population. The institutional review boards of all participating institutions approved the protocol for this retrospective study.

### 2.3. Statistical Analysis

The OS was defined as the period from the start of first-line chemotherapy until the date of death from any cause. The PFS was defined as the period from the start of first-line chemotherapy until the date of progressive disease or death from any cause. The Kaplan–Meier method was used to estimate the OS and PFS curves. The log-rank test was used to evaluate the differences among the subgroups. Cox proportional hazard models were used to adjust for potential confounding factors. All categorical variables were analyzed using Fisher’s exact test. Statistical significance was set at *p* < 0.05. All analyses were performed using JMP 16 for Windows (SAS Institute Japan Inc., Tokyo, Japan).

## 3. Results

### 3.1. Patient Characteristics

The clinical characteristics of the 286 patients with ATC are presented in Table 1. Most patients had squamous histology (66.4%). Postoperative recurrence was noted in 53 patients (18.5%). For the first-line chemotherapy regimens, platinum-based doublets, other multidrug chemotherapies, and single agents were used in 178 (62.2%), 98 (34.3%), and 10 patients (3.5%), respectively. The most popular regimens were carboplatin/paclitaxel (70 patients) among the platinum-based doublets, ADOC (79 patients) among the other multidrug chemotherapies, and S-1 (4 patients) among the single agents. S-1 is an oral fluoropyrimidine agent containing the 5-fluorouracil prodrug tegafur and two enzyme inhibitors, which can reduce the adverse effects of tegafur.

The tumor markers were assessed in 270 of the 286 patients (94.4%). In most cases, the number of tumor marker types measured was five (22.7%), with a median of four types.

### 3.2. Tumor Markers

The details of the tumor markers assessed and their median values are shown in Table 2. The most frequently assessed tumor marker before first-line chemotherapy was the CEA level (82.9%), followed by the CYFRA (73.1%), SCC antigen (66.4%), ProGRP (58.0%), NSE (51.4%), and AFP levels (37.4%). The data on the histological types limited to SCC only are also shown in Table 2.

### 3.3. Univariate Analysis of the Relationship between the OS/PFS and Each Tumor Marker

The median follow-up time was 55.5 months. The results of the comparison of the OS and PFS between the elevated-level group and the normal-level group for each tumor marker are shown in Table 3.

**Figure 1:**
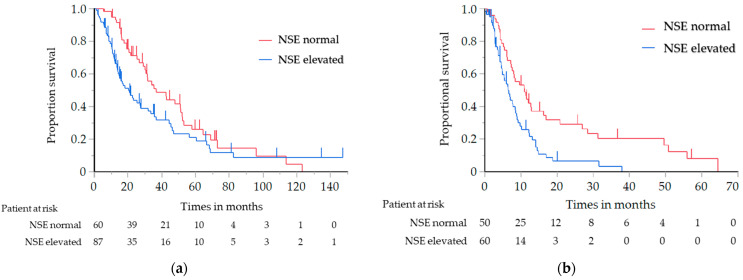
(**a**) Analysis of the overall survival in relation to the NSE level in all patients with ATC; (**b**) Analysis of the progression-free survival in relation to the NSE level in all patients with ATC. ATC, advanced thymic carcinoma; NSE, neuron-specific enolase.

The OS of the elevated NSE level group was significantly shorter than that of the normal NSE level group (median, 20.3 vs. 36.8 months; log-rank test *p* = 0.029, hazard ratio (HR), 1.55; 95% confidence interval (CI), 1.04–2.31 (Cox proportional hazard model)) (Figure 1a). The PFS with first-line chemotherapy was also significantly shorter in the elevated NSE level group than in the normal NSE level group (median, 6.4 vs. 11.0 months; log-rank test *p* = 0.001; HR, 2.04; 95% CI, 1.31–3.18) (Figure 1b). There were no significant differences in the OS and PFS among the other tumor markers. After stratification based on the NSE level, the patient characteristics were found to be well-balanced; these are summarized in Table S1.

**Figure 2:**
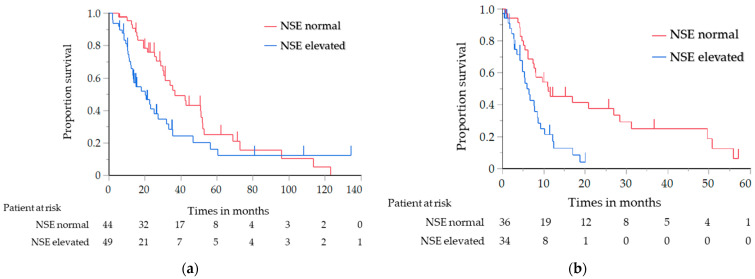
(**a**) Analysis of the overall survival in relation to the NSE level in the patients with SCC; (**b**) Analysis of the progression-free survival in relation to the NSE level in the patients with SCC. NSE, neuron-specific enolase; SCC, squamous cell carcinoma.

**Figure 3:**
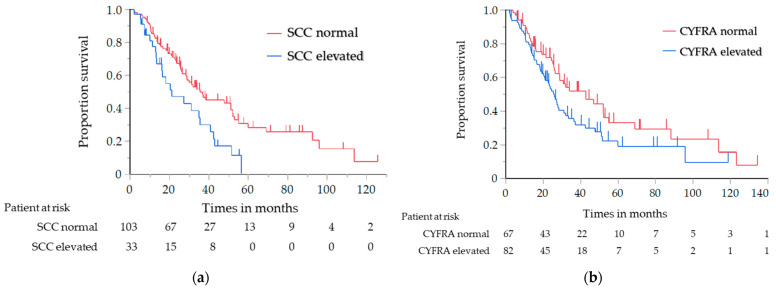
Analysis of the overall survival in the patients with SCC: (**a**) analysis in relation to the SCC antigen level, (**b**) analysis in relation to the CYFRA level. SCC, squamous cell carcinoma; CYFRA, cytokeratin-19 fragment.

The results of the analysis of the OS and PFS, in which the histological type was limited to SCC only, are shown in Table 4. Similar to the results of the previous analysis, the OS of the elevated NSE level group was significantly shorter than that of the normal NSE level group (median, 20.3 vs. 36.8 months; log-rank test *p* = 0.030; HR, 1.71; 95% CI, 1.05–2.80) (Figure 2a). The PFS with first-line chemotherapy was also significantly shorter in the elevated NSE level group than in the normal NSE level group (median, 5.9 vs. 11.0 months; log-rank test *p* = 0.001; HR, 2.52; 95% CI, 1.41–4.59) (Figure 2b). For the other tumor markers, the OS of the elevated CYFRA and SCC antigen level group was significantly shorter than that of the normal CYFRA and SCC antigen level group (CYFRA level: median, 25.7 vs. 42.7 months; log-rank test *p* = 0.047; HR, 1.52; 95% CI, 1.01–2.33; SCC antigen level: median, 21.1 vs. 33.9 months; log-rank test *p* = 0.018; HR, 1.79; 95% CI, 1.08–2.87) (Figure 3a,b); however, the PFS did not significantly differ between them.

### 3.4. Multivariate Analysis of the OS/PFS Including Factors and Tumor Markers

The results of the univariate and multivariate analyses of the OS using factors including the patient characteristics and NSE level are shown in Table 5. In the univariate analysis, the NSE level was significantly predictive of the OS (HR, 1.55; 95% CI, 1.04–2.31; *p* = 0.029). In the multivariate analysis, the NSE level was also associated with the OS (HR, 1.67; 95% CI, 1.02–2.73; *p* = 0.042). In both univariate and multivariate analyses, the NSE level was significantly predictive of the PFS (univariate: HR, 2.52; 95% CI, 1.40–4.54; *p* = 0.002; multivariate: HR, 2.24; 95% CI, 1.11–4.50; *p* = 0.024).

The results of the univariate and multivariate analyses of the OS in relation to the NSE level, limited to the patients with SCC, only are shown in Table S2. In the univariate analysis, the NSE level was significantly predictive of the OS (HR, 1.71; 95% CI, 1.05–2.80; *p* = 0.032). Further, the SCC antigen (HR, 1.95; 95% CI, 1.20–3.16; *p* = 0.007) and CYFRA levels (HR, 1.52; 95% CI, 1.00–2.32; *p* = 0.048) were also significantly correlated with the OS. In the multivariate analysis, the NSE level was associated with the OS (HR, 2.27; 95% CI, 1.21–4.27; *p* = 0.011). Under the same multivariate analysis conditions as the NSE level analysis, significant differences were observed in the SCC antigen level (HR, 2.07; 95% CI, 1.26–3.39; *p* = 0.004), but not in the CYFRA level (HR, 1.38; 95% CI, 0.88–2.17; *p* = 0.155).

In both univariate and multivariate analyses, the NSE level was significantly predictive of the PFS (univariate: HR, 2.52; 95% CI, 1.40–4.54; *p* = 0.024; multivariate: HR, 2.24; 95% CI, 1.11–4.50; *p* = 0.024), but not the SCC antigen (univariate: HR, 1.29; 95% CI, 0.79–2.10; *p* = 0.303) and CYFRA levels (univariate: HR, 1.05; 95% CI, 0.69–1.61; *p* = 0.808).

## 4. Discussion

In this study, we examined the association between tumor markers and prognosis using data from 286 patients with ATC. To our knowledge, this is the first study to investigate the relationship between tumor markers and prognosis. The results of this study indicate that the NSE level may be a prognostic factor in patients with ATC. In addition, the SCC antigen level may also be a prognostic factor in patients with SCC.

To date, many studies on the clinicopathological prognostic factors of ATC have been conducted [5,18,23]. Recently, the prognostic factors of molecular characterization, such as insulin-like growth factor-1 receptor gene amplification [39], mesothelin [40], cyclin-dependent kinases [41], programmed death ligand 1 [42,43,44], epidermal growth factor receptor [39,45], *c-KIT* [45,46], and exportin 1 [47], have been investigated. Various results regarding these factors were obtained in each study. For tumor markers, some of them were mentioned only in case reports [48,49]; however, none were investigated as prognostic factors.

In this study, a correlation was found between the NSE level and the OS and PFS. We found that the pretreatment serum NSE level was an independent prognostic factor for ATC.

The NSE level is considered to be a useful diagnostic tumor marker for tumors of neural and neuroendocrine origins, such as neuroendocrine tumors, and tends to increase in neuroendocrine carcinoma [50,51]. In this study, the NSE level was significantly higher in the patients with neuroendocrine carcinoma than in those with SCC (*p* = 0.009, Wilcoxon rank sum test). This study included 37 cases of neuroendocrine tumors, and analysis was performed excluding these cases. The results were similar to those of the entire population (data were shown in Figure S1a,b and Tables S3–S5).

Among ATCs, SCC has a better prognosis than other histological types, except for basaloid carcinoma [8]; thus, SCC is considered to have a better prognosis than neuroendocrine carcinoma. We thought that a higher NSE level in patients with ATC reflects a phenotypic heterogeneity with a larger neuroendocrine component and may be related to worse prognosis. Several reports have suggested that the NSE level may be a negative prognostic marker for non-small-cell lung cancer for the same reason [28,52,53]. NSE is a key enzyme in glycolysis and plays an important role in aerobic glycolysis [54]. Cells with a high NSE expression are thought to proliferate more rapidly, and higher NSE levels may also indicate that the cancer progresses more rapidly with a poor prognosis.

Recently, next-generation sequencing (NGS) for ATC has become a popular topic. In TC, *TP53* was one of the most frequent mutation genes, and the mutation frequency ranged from 7.7% to 26.7%; on the other hand, *TP53* mutations were rare in thymomas [55]. Several studies have suggested that *TP53* mutations are a negative prognostic factor for ATC [55,56]. *TP53* mutation seems to be associated with a more aggressive behavior, as confirmed in an earlier report [57]. Another report mentioned that in ATC, p53 immunohistochemical expression, which is correlated with *TP53* mutation, is correlated with ^18^F-FDG uptake [58]. *TP53* mutation is also thought to be associated with resistance to chemotherapy because of the involvement of multidrug resistance gene 1 (MDR1/ABCB1) [59]. Therefore, ATC with *TP53* mutations is expected to have a poor prognosis. Although their study evaluated lung adenocarcinoma, Xu et al. reported that there was a correlation between serum NSE level elevation and polygenic mutations combined with *TP53* mutations [60]. Thus, we assumed that *TP53* mutations may be seen in ATC cases with high NSE levels, which may cause a poor prognosis.

Recently, immune check inhibitors (ICIs) for ATC have gained increasing attention, and many clinical trials have been reported [61,62,63]. Giaccone et al. reported the results of a phase II trial, in which pembrolizumab was administered to patients with ATC and NGS was performed. The most commonly mutated gene was *TP53* (13 (36%) of 36 patients), and there was a correlation found between *TP53* mutations and a shorter OS [61]. Although *TP53* mutations may reduce the effect of ICIs in thymic cancer, there are reports of the opposite in lung cancer [64]; thus, further studies investigating the relationship with the NSE level are required.

The limitation of this study is its retrospective design without preregulation to measure the tumor markers, and a selection bias could have occurred. The generally permitted missing data ratio is less than 5%, and there were data losses more than 5% in this study, which may have affected the results. Since many tumor markers were examined, there was also a problem of statistical multiplicity. Moreover, there was an unbalanced number of cases between the high and normal levels of tumor markers other than the NSE and CYFRA levels. Therefore, it might be difficult to observe a significant difference in these tumor markers. Next, TC was diagnosed in each institution, and a central pathological judgment was absent. The previous reports mentioned that pathological diagnosis of thymic epithelial tumor is difficult, and the diagnosis concordance rate among pathologists is not high [10,65]. For this study, histological diagnosis should have been performed centrally, but this was difficult to carry out because some pathological specimens used for diagnosis were no longer stored, and were not available. The diagnoses and histological subtypes may not be as accurate as those in studies with a central pathological judgment, although this study was conducted at institutions with sufficient skills for the diagnosis and treatment of ATC. As some of tissue specimens were old and not stored any longer, the levels of NSE in tumor samples were not evaluated.

## 5. Conclusions

This study investigated whether tumor markers are useful prognostic factors in patients with ATC. The NSE level was found to be a prognostic factor for all histological types of ATC, while the SCC antigen level was found to be a prognostic factor for the SCC type.

## Figures and Tables

**Table 1:** Materials’ characteristics (*n* = 286).

Characteristics	*n* (%)
Age, median(range), y	61 (13–84)
Sex, male/female	137/54 (71.7/28.3)
Eastern Cooperative Oncology Group performance status	
0–1	248 (86.7)
2–3	146 (10.8)
Unknown	7 (2.4)
Histology	
Squamous cell carcinoma	190 (66.4)
Undifferentiated carcinoma	30 (10.5)
Poorly differentiated neuroendocrine carcinoma	29 (10.1)
Well-differentiated neuroendocrine carcinoma	8 (2.8)
Adenocarcinoma	4 (1.4)
Mucoepidermoid carcinoma	2 (0.7)
Sarcomatoid carcinoma	2 (0.7)
Papillary adenocarcinoma	1 (0.5)
Lymphoepithelioma-like carcinoma	1 (0.5)
Basaloid carcinoma	1 (0.5)
Staging	
Masaoka–Koga staging	
Stage III or postoperative recurrence	69 (24.1)
Stage IVa	75 (26.2)
Stage IVb	144 (50.3)
World Health Organization TNM staging	
Stage III or postoperative recurrence	64 (22.4)
Stage IV	222 (77.6)
First-line chemotherapy regimens	
Platinum-based doublets	178 (62.2)
Other multidrug chemotherapies	98 (34.3)
Single agents	10 (3.5)
Number of tumor marker types assessed	
6	41 (14.3)
5	65 (22.7)
4	56 (19.6)
3	56 (19.6)
2	38 (13.3)
1	14 (4.9)
0	16 (5.6)

**Table 2:** Tumor markers and number of patients assessed.

Tumor Marker	*n* (%)	Median (Range)
All patients (*n* = 286)		
CEA level (normal range ≤ 5.0 ng/mL)	237 (82.9)	2.1 (0.2–182.8) ng/mL
CYFRA level (normal range ≤ 3.5 ng/mL)	209 (73.1)	3.8 (0.4–150) ng/mL
SCC antigen level (normal range ≤ 1.5 ng/mL)	190 (66.4)	0.95 (0.2–70) ng/mL
ProGRP level (normal range ≤ 80 pg/mL)	166 (58.0)	27.6 (4.0–1890) pg/mL
NSE level (normal range ≤ 10 ng/mL)	147 (51.4)	11.8 (1.9–231.4) ng/mL
AFP level (normal range ≤ 10 ng/mL)	107 (37.4)	3.2 (1.0–40) ng/mL
Patients with SCC (*n* = 190)		
CEA level	167 (87.9)	2.0 (0.2–83.5) ng/mL
CYFRA level	149 (78.4)	4.4 (0.5–150) ng/mL
SCC antigen level	136 (71.6)	1.0 (0.2–70) ng/mL
ProGRP level	103 (54.2)	28.9 (4.0–97.6) pg/mL
NSE level	93 (48.9)	10.4 (1.9–113.2) ng/mL
AFP level	76 (40.0)	3.05 (1.0–26.3) ng/mL

CEA, carcinoembryonic antigen; CYFRA, cytokeratin-19 fragment; SCC, squamous cell carcinoma; ProGRP, progastrin-releasing peptide; NSE, neuron-specific enolase; AFP, alpha-fetoprotein.

**Table 3:** Univariate analysis of the relationship between the OS/PFS and each tumor marker (all patients [*n* = 286]).).

Tumor Marker		OS			PFS		
	*n* (%)	Median (95% CI)	HR (95% CI) *	*p* Value ^†^	Median (95% CI)	HR (95% CI) *	*p* Value ^†^
CEA level (*n* = 237)							
Normal	202 (85.2)	31.2 (25.9–37.1)	1		8.5 (7.1–9.8)	1	
Elevated	35 (14.8)	25.6 (11.3–40.8)	1.57 (0.99–2.51)	0.054	6.2 (4.3–9.2)	1.42 (0.85–2.37)	0.172
CYFRA level (*n* = 209)							
Normal	100 (47.8)	34.9 (26.3–48.2)	1		8.5 (7.0–11.7)	1	
Elevated	109 (52.1)	24.3 (18.4–31.0)	1.31 (0.93–1.85)	0.123	8.4 (6.0–9.2)	0.96 (0.67–1.36)	0.814
SCC antigen level (*n* = 190)							
Normal	145 (76.3)	33.9 (25.7–45.4)	1		8.1 (7.0–9.0)	1	
Elevated	45 (23.7)	27.2 (16.3–39.4)	1.31 (0.87–1.99)	0.194	9.6 (7.8–12.8)	0.84 (0.55–1.28)	0.491
ProGRP level (*n* = 166)							
Normal	157 (94.6)	26.5 (18.4–35.5)	1		7.4 (6.2–8.8)		
Elevated	9 (5.4)	27.8 (3.7–82.3)	0.94 (0.41–2.15)	0.885	14.1 (3.7–27.8)	0.82 (0.33–2.02)	0.662
NSE level (*n* = 147)							
Normal	60 (40.8)	36.8 (29.9–51.9)			11.0 (7.3–16.2)	1	
Elevated	87 (59.2)	20.3 (13.9–31.9)	1.55 (1.04–2.31)	0.029	6.4 (4.6–8.6)	2.04 (1.31–3.18)	0.001
AFP level (*n* = 107)							
Normal	101 (94.4)	28.9 (18.1–45.4)	1		8.0 (5.8–9.0)	1	
Elevated	6 (5.6)	33.4 (15.7–45.4)	1.04 (0.33–3.34)	0.945	4.8 (1.6–NE)	2.60 (0.91–7.440)	0.065

OS, overall survival; PFS, progression-free survival; HR, hazard ratio; CI, confidence interval; CEA, carcinoembryonic antigen; CYFRA, cytokeratin-19 fragment; SCC, squamous cell carcinoma; ProGRP, progastrin-releasing peptide; NSE, neuron-specific enolase; AFP, alpha-fetoprotein; NE, not estimated. * Cox hazard method, ^†^ log-rank test.

**Table 4:** Univariate analysis of the relationship between the OS/PFS and each tumor marker (patients with SCC [*n* = 190]).

Tumor Marker		OS			PFS		
	*n* (%)	Median (95% CI)	HR (95% CI) *	*p* value ^†^	Median (95% CI)	HR (95% CI) *	*p* Value ^†^
CEA level (*n* = 167)							
Normal	147 (88.0)	31.9 (26.3–42.7)	1		8.5 (7.3–9.9)	1	
Elevated	20 (12.0)	28.9 (10.3–42.4)	1.52 (0.83–2.80)	0.175	6.2 (3.7–10.1)	1.32 (0.70–2.48)	0.387
CYFRA level (*n* = 149)							
Normal	67(45.0)	42.7 (26.3–52.2)	1		9.2 (7.2–12.4)	1	
Elevated	82 (55.0)	25.7 (13.4–35.4)	1.52 (1.00–2.32)	0.047	8.4 (5.8–9.8)	1.05 (0.69–1.61)	0.808
SCC antigen level (*n* = 136)							
Normal	103 (75.7)	35.5 (28.3–52.0)	1		8.4 (7.2–10.9)	1	
Elevated	33 (24.3)	21.1 (13.4–35.4)	1.95 (1.20–3.16)	0.006	8.6 (4.1–10.2)	1.29 (0.79–2.10)	0.301
ProGRP level (*n* = 103)							
Normal	101(98.1)	27.2 (20.8–37.1)	1		7.6 (6.2–9.6)	1	
Elevated	2 (1.9)	21.0 (14.2–27.8)	1.95 (0.47–7.91)	0.358	27.8 (NE–NE)	0.55 (0.08–4.02)	0.551
NSE level (*n* = 93)							
Normal	44 (47.3)	36.8 (29.9–51.9)	1		11.0 (7.3–28.4)	1	
Elevated	49 (52.7)	20.3 (13.2–27.2)	1.71 (1.05–2.80)	0.030	5.9 (4.2–8.4)	2.52 (1.40–4.54)	0.001
AFP level (*n* = 76)							
Normal	73 (96.1)	28.9 (16.6–42.8)	1		8.1 (5.8–9.2)	1	
Elevated	3 (3.9)	45.4 (NE–NE)	0.53 (0.07–3.88)	0.527	4.8 (1.6–4.9)	4.12 (1.20–14.18)	0.015

OS, overall survival; PFS, progression-free survival; HR, hazard ratio; CI, confidence interval; CEA, carcinoembryonic antigen; CYFRA, cytokeratin-19 fragment; SCC, squamous cell carcinoma; ProGRP, progastrin-releasing peptide; NSE, neuron-specific enolase; AFP, alpha-fetoprotein; NE, not estimated. * Cox hazard method, ^†^ log-rank test.

**Table 5:** Univariate and multivariate analyses of the overall survival including the NSE level and patient background (all patients [*n* = 286]).

Category	*N*	Median (95% CI) (Months)	Univariate		Multivariate	
			HR (95% CI)	*p* Value	HR (95% CI)	*p* Value
NSE level						
Normal	60	36.8 (29.9–51.9)	1		1	
Elevated	87	20.3 (13.9–31.9)	1.55 (1.04–2.31)	0.030	1.67 (1.02–2.73)	0.042
Age (y)						
<65	182	30.5 (23.2–36.7)	1		1	
≥65	104	31.2 (25.7–37.1)	1.03 (0.76–1.39)	0.853	0.74 (0.45–1.22)	0.241
Sex						
Female	84	21.3 (16.4–35.5)	1		1	
Male	202	31.9 (27.2–40.8)	0.71 (0.53–0.97)	0.031	0.84 (0.52–1.35)	0.463
Eastern Cooperative Oncology Group performance status						
0–1	248	32.0 (27.8–37.9)	1		1	
2–3	31	17.7 (11.3–21.3)	1.75 (1.13–2.72)	0.012	1.58 (0.83–3.00)	0.162
Histology						
Squamous cell carcinoma	190	31.9 (27.2–38.3)	1		1	
Neuroendocrine carcinoma	37	27.0 (16.3–45.0)	1.35 (0.89–2.06)	0.157	0.67 (0.37–1.20)	0.174
Others	59	21.3 (14.8–35.9)	1.32 (0.92–1.89)	0.137	0.67 (0.36–1.20)	0.176
Masaoka stage						
Recurrence/III	67	36.5 (28.9–51.7)	1		1	
IVa	75	42.8 (28.2–52.9)	0.82 (0.54–1.26)	0.365	0.91 (0.20–4.16)	0.907
IVb	144	21.3 (16.3–28.5)	1.72 (1.19–2.48)	0.004	2.16 (0.50–9.21)	0.299
(IVb vs. IVa)			2.09 (1.47–2.99)	<0.001	2.36 (1.37–4.07)	0.002
World Health Organization TNM stage						
Recurrence/III	64	38.3 (30.5–51.9)	1		1	
IV	222	27.2 (23.2–33.9)	1.37 (0.96–1.99)	0.087	0.39 (0.09–1.75)	0.281
Volume-reduction surgery						
Yes	23	52.0 (28.5–123.2)	1		1	
No	263	28.9 (24.4–34.9)	2.13 (1.19–3.84)	0.012	1.50 (0.62–3.62)	0.366
Volume-reduction radiotherapy						
Yes	47	42.4 (32.0–52.2)	1		1	
No	240	27.2 (23.9–31.9)	1.43 (0.96–2.21)	0.093	1.47 (0.82–2.62)	0.196
First-line chemotherapy regimen						
Platinum-based doublet	178	30.7 (24.5–37.9)	1			
Monotherapy	10	54.9 (1.1–95.9)	0.73 (0.34–1.58)	0.431	0.50 (0.13–1.84)	0.300
Other multidrug regimens	98	29.9 (23.2–37.1)	0.94 (0.70–1.27)	0.702	0.92 (0.58–1.47)	0.735

HR, hazard ratio; CI, confidence interval; NSE, neuron-specific enolase. Cox hazard method.

## Data Availability

All data presented in this study are available in this article and Supplementary Material.

## Supplementary Materials

The following are available online at https://www.mdpi.com/article/10.3390/cancers14020331/s1, Figure S1 (a): Analysis of the overall survival in relation to the NSE level in patients with ATC excluding neuroendocrine tumors, (b): Analysis of the progression-free survival in relation to the NSE level in patients with ATC excluding neuroendocrine tumors, Table S1: Patient characteristics according to the NSE level (all patients (*n* = 286)), Table S2: Univariate and multivariate analyses of the overall survival including the NSE level and patient background (patients with squamous cell carcinoma (*n* = 190)),Table S3: Tumor markers and number of patients except for neuroendocrine tumors assessed, Table S4: Univariate analysis of the relationship between the OS/PFS and each tumor marker (patients except for neuroendocrine tumors (*n* = 249)), Table S5: Univariate and multivariate analysis for overall survival in neuron-specific enolase (NSE) and patient background (patients except for neuroendocrine tumors (*n* = 249)).
