# Supplementary material for: Clinical Significance of Tumor Markers for Advanced Thymic Carcinoma: A Retrospective Analysis from the NEJ023 Study
Source: Cancers (Basel). 2022 Jan 11;14(2):331. doi: 10.3390/cancers14020331 (PMC8773938; doi:10.3390/cancers14020331)
Supplement: Supplementary file 1 [file cancers-14-00331-s001.zip › cancers-1497953-supplementary.pdf]

Supplementary materials: Clinical Significance of Tumor Markers for Advanced Thymic Carcinoma: A Retrospective Analysis from the NEJ023 Study

Tomoyasu Mimori, Takehito Shukuya, Ryo Ko, Yusuke Okuma, Tomonobu Koizumi, Hisao Imai, Yuichi Takiguchi, Eisaku Miyauchi, Hiroshi Kagamu, Tomohide Sugiyama, Keisuke Azuma, Yukiko Namba, Masahiro Yamasaki, Hisashi Tanaka, Yuta Takashima, Sayo Soda, Osamu Ishimoto, Nobuyuki Koyama, Kunihiko Kobayashi and Kazuhisa Takahashi

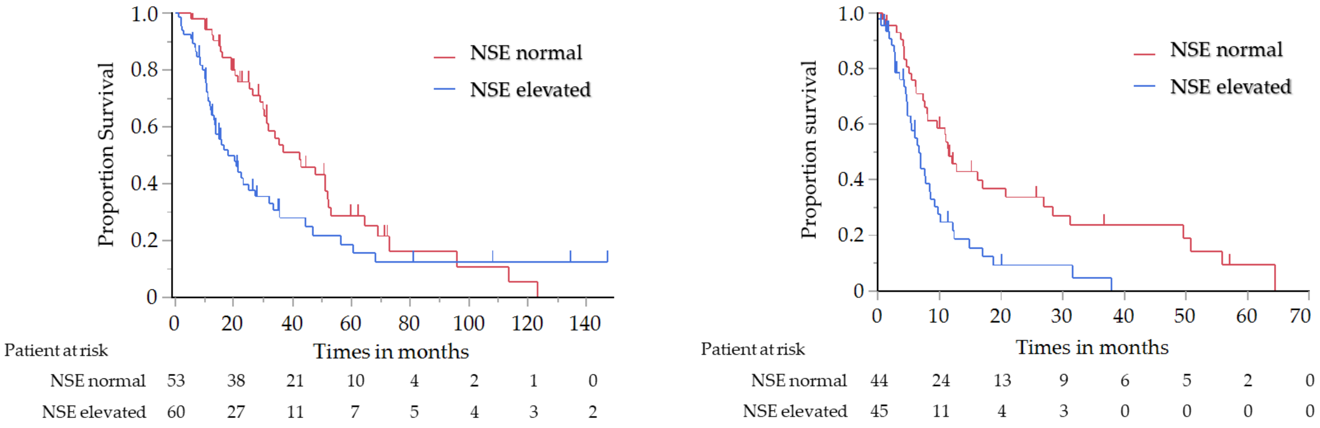

a b

Figure S1. (**a**) Analysis of the overall survival in relation to the NSE level in patients with ATC excluding neuroendocrine tumors; (**b**) Analysis of the progression-free survival in relation to the NSE level in patients with ATC excluding neuroendocrine tumors. ATC, advanced thymic carcinoma; NSE, neuron-specific enolase.

Table S1. Patient characteristics according to neuron-specific enolase (NSE) levels (all patients [*n* = 286]).

Category	**NSE Level Normal** [*n* = 60]	**NSE Level Elevated** [*n* = 87]	**Balance**
	***n* (%)**	***n* (%)**	***p* Value***
Age			
<65	38 (63.3)	29 (33.3)	
≧65	22 (36.7)	58 (66.7)	0.726
Sex			
Female	14 (23.3)	34 (39.1)	
Male	46 (77.7)	53 (60.9)	0.051
ECOG PS			
0–1	49 (83.1)	75 (89.3)	
2–3	10 (17.0)	9 (10.7)	0.322
Histology			
SCC	44 (73.3)	49 (56.3)	
NEC	7 (11.7)	21 (24.1)	
Others	9 (15.0)	17 (19.5)	0.086
Masaoka TNM Stage			
recurrence/III	13 (21.7)	11 (12.6)	
IVa	17 (28.3)	20 (23.0)	
IVb	30 (50.0)	56 (64.4)	0.189

WHO TNM Stage			
recurrence/III	12 (20.0)	8 (9.2)	
IV	48 (80.0)	79 (90.8)	0.086
Volume reduction surgery			
Yes	53 (88.3)	83 (95.4)	
No	7 (11.7)	4 (4.6)	0.124
Volume reduction radiotherapy			
Yes	50 (83.3)	70 (80.5)	
No	10 (16.7)	17 (19.5)	0.829
First–line chemotherapy regimen			
Platinum doublet	32 (53.3)	60 (69.0)	
Monotherapy	4 (6.7)	25 (28.7)	
Other multidrug regimens	24 (40.0)	2 (2.3)	0.133

Table S2. Univariate and multivariate analysis for overall survival in neuron–specific enolase (NSE) and patient background (squamous cell carcinoma [*n* = 190]).

Category	*n*	Median(95%CI) (Months)	Univariate		Multivariate	
			HR (95%CI)	*p* Value	HR (95%CI)	*p* Value
NSE level						
Normal	44	36.8 (29.9–51.9)	1		1	
Elevated	49	20.3 (13.9–27.2)	1.71 (1.05–2.80)	0.032	2.27 (1.21–4.27)	0.011
Age						
<65	114	31.9 (25.3–45.4)	1		1	
≧65	76	32.0 (26.3–40.8)	1.13 (0.78–1.62)	0.526	0.94 (0.49–1.79)	0.847
Sex						
Female	56	30.5 (16.4–38.3)	1		1	
Male	134	33.3 (27.8–45.4)	0.72 (0.49–1.05)	0.086	1.06 (0.56–2.01)	0.854
ECOG PS						
0–1	170	33.9 (28.9–42.7)	1		1	
2–3	14	18.1 (10.1–35.5)	1.56 (0.81–3.00)	0.180	1.76 (0.76–4.08)	0.189
Masaoka TNM Stage						
recurrence/III	47	38.3 (28.9–51.9)	1		1	
IVa	53	52.9 (31.9–NE)	0.56 (0.33–0.96)	0.036	0.53 (0.06–4.46)	0.559
IVb	90	23.2 (15.6–31.0)	1.73 (1.12–2.67)	0.014	2.02 (0.26–15.83)	0.504
(IVb vs. IVa)			3.06 (1.91–4.91)	<0.001	3.81 (1.87–7.77)	<0.001
WHO TNM Stage						
recurrence/III	46	38.3 (28.9–54.9)	1		1	
IV	144	31.0 (25.1–36.8)	1.22 (0.79–1.87)	0.373	0.45 (0.05–3.86)	0.468
Volume reduction surgery						
Yes	15	52.0 (29.9–123.2)	1			
No	175	31.0 (25.9–36.5)	2.00 (0.97–4.12)	0.059	1.20 (0.44–3.26)	0.718
Volume reduction radiotherapy						
Yes	31	46.8 (32.0–60.6)	1			
No	159	30.5 (25.6–36.8)	1.64 (0.97–2.78)	0.066	2.13 (1.01–4.51)	0.047
First–line chemotherapy regimen						
Platinum doublet	116	31.9 (25.6–45.4)	1			
Monotherapy	8	54.9 (26.3–95.9)	0.59 (0.24–1.46)	0.253	0.33 (0.07–1.60)	0.171
Other multidrug regimens	66	31.9 (24.4–42.4)	0.93 (0.64–1.36)	0.725	0.84 (0.47–1.47)	0.537

HR; hazard ratio, CI; confidential interval, NSE; neuron-specific enolase, ECOG; Eastern Cooperative Oncology Group, PS; performance status, NE; not estimated, WHO; World Health Organization.

Table S3. Tumor markers and number of patients except for neuroendocrine tumors assessed.

Tumor Marker	*n* (%)	Median (Range)

Patients except for neuroendocrine tumors (*n* = 249)		
CEA level	212 (85.1)	2.1 (0.2–182.8) ng/mL
CYFRA level	187 (75.1)	4.2 (0.4–150) ng/mL
SCC antigen level	171 (68.7)	1 (0.2–70) ng/mL
ProGRP level	139 (55.8)	27.4 (4–188) pg/mL
NSE level	119 (47.8)	10.9 (1.9–231.4) ng/mL
AFP level	94 (37.8)	3.1 (1.0–40) ng/mL

CEA, carcinoembryonic antigen; CYFRA, cytokeratin-19 fragment; SCC, squamous cell carcinoma; ProGRP, pro-gastrin-releasing peptide; NSE, neuron-specific enolase; AFP, alpha-fetoprotein.

Table S4. Univariate analysis of the relationship between the OS/PFS and each tumor marker (patients except for neuroendocrine tumors [*n* = 249]).

Tumor Marker		OS			PFS		
	n (%)	Median (95% CI)	HR (95% CI)*	*p* Value†	Median (95% CI)	HR (95% CI)*	*p* Value†
CEA level (*n* = 212)							
Normal	182 (85.8)	31.7 (25.9–38.3)	1		8.6 (7.3–10.9)	1	
Elevated	30 (14.2)	28.9 (10.3–40.8)	1.64 (0.98–2.86)	0.058	6.2 (3.7–9.2)	1.53 (0.87–2.68)	0.136
CYFRA level (*n* = 187)							
Normal	86 (47.8)	42.7 (26.3–51.9)	1		9.2 (7.0–12.4)	1	
Elevated	101 (52.1)	25.3 (19.6–31.9)	1.32 (0.91–1.90)	0.142	8.4 (5.8–9.2)	1.04 (0.71–1.51)	0.847
SCC antigen level (*n* = 171)							
Normal	129 (75.4)	33.9 (25.7–44.3)	1		8.1 (7.0–9.8)	1	
Elevated	42 (24.6)	27.2 (16.0–40.8)	1.30 (0.85–2.01)	0.194	9.2 (4.8–12.5)	1.00 (0.65–1.53)	0.994
ProGRP level (*n* = 139)							
Normal	136 (97.8)	27.2 (20.4–37.1)	1		7.6 (6.2–9.6)		
Elevated	3 (2.2)	27.8 (14.2–NE)	0.97 (0.25–4.18)	0.973	27.8 (NR–NR)	0.63 (0.09–4.53)	0.639
NSE level (*n* = 119)							
Normal	53 (44.5)	42.4 (30.5–51.9)			11.5 (7.6–20.8)	1	
Elevated	66 (55.5)	18.1 (13.2–27.2)	1.72 (1.11–2.67)	0.015	6.6 (4.8–8.6)	2.19 (1.33–3.61)	0.002
AFP level (*n* = 94)							
Normal	88 (93.6)	28.9 (16.6–46.8)	1		8.0 (5.4–9.0)	1	
Elevated	6 (6.4)	33.4 (15.7–45.4)	0.99 (0.31–3.19)	0.989	4.8 (1.6–NE)	2.60 (0.90–7.52)	0.066

OS, overall survival; PFS, progression-free survival; HR, hazard ratio; CI, confidence interval; CEA, carcinoembryonic antigen; CYFRA, cytokeratin-19 fragment; SCC, squamous cell carcinoma; ProGRP, pro-gastrin-releasing peptide; NSE, neuron-specific enolase; AFP, alpha-fetoprotein; NE, not estimated. *Cox hazard method, †log-rank test.

Table S5. Univariate and multivariate analysis for overall survival in neuron-specific enolase (NSE) and patient background (patients except for neuroendocrine tumors [*n* = 249]).

Category	*n*	Median(95%CI) (Months)	Univariate		Multivariate	
			HR (95%CI)	*p* Value	HR (95%CI)	*p* Value
NSE level						
Normal	53	42.4 (30.5–51.9)	1		1	
Elevated	66	18.1 (13.2–27.2)	1.72 (1.11–2.67)	0.016	1.86 (1.08–3.21)	0.026
Age						
<65	157	30.5 (23.2–38.3)	1		1	
≧65	92	31.2 (25.9–37.1)	1.06 (0.76–1.47)	0.731	0.94 (0.53–1.66)	0.826
Sex						

Female	72	16.5 (13.8–21.3)	1		1	
Male	177	25.3 (21.4–27.8)	0.67 (0.48–0.94)	0.019	0.77 (0.45–1.32)	0.344
ECOG PS						
0–1	218	32.0 (28.5–40.8)	1		1	
2–3	24	15.0 (10.1–19.2)	2.02 (1.23–3.30)	0.006	1.70 (0.84–3.44)	0.141
Masaoka TNM Stage						
recurrence/III	56	36.5 (28.9–51.7)	1		1	
IVa	68	48.2 (31.9–68.2)	0.75 (0.48–1.20)	0.232	0.37 (0.08–1.69)	0.200
IVb	125	21.3 (16.3–28.5)	1.77 (1.20–2.64)	0.005	0.96 (0.22–4.18)	0.962
(IVb vs. IVa)			2.35 (1.59–3.47)	<0.001	2.61 (1.46–4.69)	0.001
WHO TNM Stage						
recurrence/III	54	38.3 (30.5–54.9)	1		1	
IV	195	27.8 (23.5–33.9)	1.42 (0.96–2.11)	0.079	0.91 (0.18–4.54)	0.826
Volume reduction surgery						
Yes	19	69.0 (29.9–123.2)	1			
No	230	28.9 (24.5–34.9)	2.47 (1.26–4.85)	0.009	1.58 (0.60–4.15)	0.350
Volume reduction radiotherapy						
Yes	38	44.3 (32.0–60.6)	1			
No	211	27.8 (24.3–33.3)	1.61 (1.01–2.57)	0.047	1.58 (0.79–3.15)	0.192
First-line chemotherapy regimen						
Platinum doublet	148	30.7 (24.5–40.8)	1			
Monotherapy	10	54.9 (1.15–95.9)	0.78 (0.36–1.68)	0.523	0.52 (0.14–1.94)	0.330
Other multidrug regimens	91	29.9 (23.5–37.1)	0.96 (0.69–1.33)	0.800	1.00 (0.60–1.67)	0.989

HR, hazard ratio; CI, confidence interval; NSE, neuron-specific enolase. *Cox hazard method.
